# Supplementary figures and images for: The association between water hardness and xerosis—Results from the Danish Blood Donor Study
Source: PLoS One. 2021 Jun 2;16(6):e0252462. doi: 10.1371/journal.pone.0252462 (PMC8171951; doi:10.1371/journal.pone.0252462)

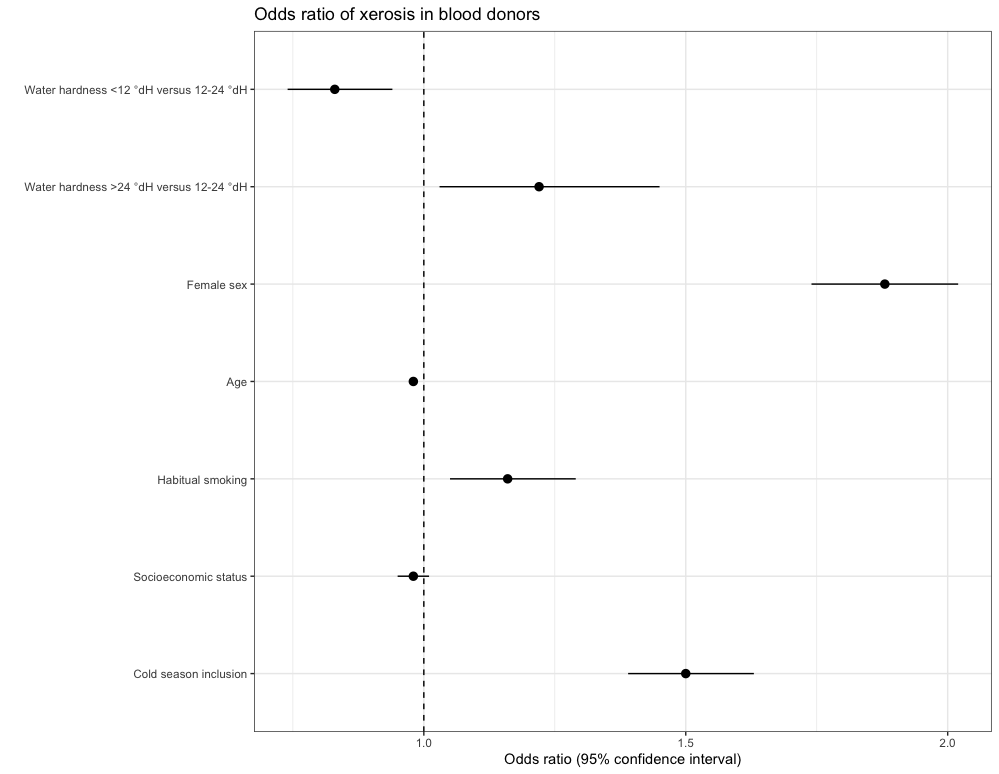

Supplement: S1 Fig — (TIFF) [file pone.0252462.s001.tiff]
