# Supplementary material for: The association between water hardness and xerosis—Results from the Danish Blood Donor Study
Source: PLoS One. 2021 Jun 2;16(6):e0252462. doi: 10.1371/journal.pone.0252462 (PMC8171951; doi:10.1371/journal.pone.0252462)
Supplement: S1 Table — (DOCX) [file pone.0252462.s002.docx]

**S1 Table. ICD-10^th^ diagnoses used to define xerosis, dermatitis, ichthyosis, lichen planus and psoriasis**

| **Xerosis** | **Dermatitis** | | | **Ichthyosis** | **Lichen planus** | **Psoriasis** |
| --- | --- | --- | --- | --- | --- | --- |
| L853 | DL20 | DL239A | DL247 | DL850 | DL43 | DL40 |
|  | DL200 | DL23 | DL247A | DQ800 | DL430 | DL400 |
|  | DL208 | DL230 | DL248 | DQ801 | DL431 | DL400B |
|  | DL208B | DL230A | DL248A | DQ802 | DL433 | DL400D |
|  | DL208C | DL230B | DL249 | DQ808B | DL438C | DL404 |
|  | DL208D | DL230C | DL249A | DQ808C | DL439 | DL409 |
|  | DL209 | DL230D | DL25 |  | DL438 | BNHC |
|  | DL21 | DL230E | DL250 |  |  | BNHC0 |
|  | DL210 | DL231 | DL251 |  |  | BNHC1 |
|  | DL210B | DL231A | DL252 |  |  | MD05 |
|  | DL210C | DL232 | DL253 |  |  | MD05BB |
|  | DL210D | DL232A | DL253A |  |  | DL400C |
|  | DL210E | DL232B | DL253B |  |  | DL401 |
|  | DL211 | DL232C | DL254 |  |  | DL408 |
|  | DL211A | DL232D | DL255 |  |  | DL408C |
|  | DL218 | DL232E | DL258 |  |  | MD05A |
|  | DL218A | DL233 | DL259 |  |  | MD05AX |
|  | DL218B | DL233A | DL259A |  |  | MD05B |
|  | DL218D | DL234 | DL26 |  |  | MD05BX |
|  | DL218E | DL234A | DL269 |  |  | DO997A |
|  | DL218F | DL235 | DL269B |  |  |  |
|  | DL219 | DL235A | DL30 |  |  |  |
|  | DL22 | DL235B | DL300 |  |  |  |
|  | DL229 | DL24 | DL301 |  |  |  |
|  | DL229B | DL240 | DL301A |  |  |  |
|  | DL235B | DL240A | DL301B |  |  |  |
|  | DL235C | DL241 | DL301C |  |  |  |
|  | DL235D | DL241A | DL302 |  |  |  |
|  | DL235E | DL242 | DL302A |  |  |  |
|  | DL235F | DL242A | DL303 |  |  |  |
|  | DL235G | DL243 | DL304 |  |  |  |
|  | DL235H | DL243A | DL305 |  |  |  |
|  | DL235I | DL244 | DL305A |  |  |  |
|  | DL236 | DL244A | DL308 |  |  |  |
|  | DL236A | DL245 | DL308A |  |  |  |
|  | DL237 | DL245A | DL308B |  |  |  |
|  | DL237A | DL245B | DL308D |  |  |  |
|  | DL238 | DL245C | DL308F |  |  |  |
|  | DL238A | DL245D | DL308G |  |  |  |
|  | DL238B | DL245E | DL308H |  |  |  |
|  | DL238C | DL246 | DL308I |  |  |  |
|  | DL239 | DL246A | DL309 |  |  |  |

ICD-10^th^, International Classification of Disease-10;
