# Supplementary material for: The association between water hardness and xerosis—Results from the Danish Blood Donor Study
Source: PLoS One. 2021 Jun 2;16(6):e0252462. doi: 10.1371/journal.pone.0252462 (PMC8171951; doi:10.1371/journal.pone.0252462)
Supplement: S2 Table — (DOCX) [file pone.0252462.s003.docx]

**S2 Table. Adjusted multivariable nominal regression with xerosis as outcome**

|  | Xerosis 1 versus Control 1 | | Xerosis 2 versus Control 2 | |
| --- | --- | --- | --- | --- |
|  | Adjusted^a^ | | Adjusted^a^ | |
|  | OR (95% CI) | P-value | OR (95% CI) | P-value |
| Water hardness <12 °dH | 0.83 (0.74–0.94) | 0.003^b^ | 0.86 (0.76–0.98) | 0.02^b^ |
| Water hardness 12-24 °dH | Ref. | Ref. | Ref. | Ref. |
| Water hardness >24 °dH | 1.22 (1.04–1.45) | 0.02^b^ | 1.21 (1.02–1.45) | 0.03^b^ |
| Age | 0.98 (0.98–0.98) | <0.001^b^ | 0.98 (0.98–0.98) | <0.001^b^ |
| Female Sex | 1.87 (1.74–2.02) | <0.001^b^ | 1.89 (1.75–2.04) | <0.001^b^ |
| Smoking | 1.16 (1.05–1.29) | 0.01^b^ | 1.16 (1.04–1.29) | 0.01^b^ |
| Cold season | 1.51 (1.39–1.63) | <0.001^b^ | 1.57 (1.44–1.70) | <0.001^b^ |

CI, Confidence Level; °dH, Degree Deutsche Härte; OR, Odds Ratio; Ref., Reference Group;

^a^Adjusted for Age, Sex, Smoking, and Cold season

^b^significant after Benjamini–Hochberg correction with a false discovery rate of 0.05%
