# Supplementary material for: The association between water hardness and xerosis—Results from the Danish Blood Donor Study
Source: PLoS One. 2021 Jun 2;16(6):e0252462. doi: 10.1371/journal.pone.0252462 (PMC8171951; doi:10.1371/journal.pone.0252462)
Supplement: S3 Table — (DOCX) [file pone.0252462.s004.docx]

**S3 Table. Multivariable nominal regression with xerosis as outcome and interaction for age and sex**

| Model: Xerosis ~ water hardness + age + sex + age*sex + smoking + Socioeconomic status + Cold season | | | | | | | | |
| --- | --- | --- | --- | --- | --- | --- | --- | --- |
|  | Xerosis 1 versus Control 1 | | | | Xerosis 2 versus Control 2 | | | |
|  | Est | SE | OR (95% CI) | P-value | Est | SE | OR (95% CI) | P-value |
| Water hardness <12 °dH | -0.18 | 0.06 | 0.83 (0.74–0.94) | 0.003^b^ | -0.15 | 0.06 | 0.86 (0.76–0.98) | 0.02^b^ |
| Water hardness 12-24 °dH | Ref. | Ref. | Ref. | Ref. | Ref. | Ref. | Ref. | Ref. |
| Water hardness >24 °dH | 0.20 | 0.09 | 1.22 (1.03–1.45) | 0.02^b^ | 0.19 | 0.09 | 1.21 (1.02–1.45) | 0.03^b^ |
| Age*Sex | 0.006 | 0.003 | 1.00 (0.99-1.01) | 0.84 | -0.0001 | 0.003 | 1.00 (0.99–1.01) | 0.97 |
| Age | -0.02 | 0.005 | 0.98 (0.97–0.99) | <0.001^b^ | -0.02 | 0.005 | 0.98 (0.97–0.99) | <0.001^b^ |
| Sex | 0.61 | 0.12 | 1.84 (1.44–2.34) | <0.001^b^ | 0.64 | 0.13 | 1.90 (1.48–2.45) | <0.001^b^ |
| Smoking | 0.15 | 0.05 | 1.16 (1.05–1.29) | 0.004^b^ | 0.15 | 0.06 | 1.16 (1.04–1.29) | 0.01^b^ |
| Socioeconomic status | -0.02 | 0.02 | 0.98 (0.95-1.01) | 0.23 | -0.02 | 0.02 | 0.98 (0.95–1.02) | 0.33 |
| Cold season | 0.41 | 0.04 | 1.50 (1.39–1.63) | <0.001^b^ | 0.45 | 0.04 | 1.56 (1.44–1.70) | <0.001^b^ |

CI, Confidence Level; °dH, Degree Deutsche Härte; Est, Estimate; OR, Odds Ratio; Ref., Reference Group; SE, Standard error;

^b^significant after Benjamini–Hochberg correction with a false discovery rate of 0.05%
